# Incidence and trends of patient MACE outcomes after Transcatheter Aortic Valve Implantation (TAVI): analysis by age and sex

**DOI:** 10.1007/s12471-025-02006-6

**Published:** 2025-12-17

**Authors:** Tsvetan R. Yordanov, Hatem Al-Farra, Anita C. J. Ravelli, Saskia Houterman, Bas AJM de Mol, Toon A. Winkelman, Leo Timmers, Marije Vis, Pim Tonino, Ronak Delewi, Ameen Abu-Hanna, José P. S. Henriques

**Affiliations:** 1 Department of Medical Informatics, Amsterdam UMC, location University of Amsterdam, Amsterdam, The Netherlands; 2 Amsterdam Public Health, Amsterdam, The Netherlands; 3 Netherlands Heart Registration, Utrecht, The Netherlands; 4 Heart Centre, Amsterdam Cardiovascular Sciences, Amsterdam UMC, University of Amsterdam, Amsterdam, The Netherlands; 5 Department of Cardiothoracic Surgery, Amsterdam UMC, Amsterdam, The Netherlands; 6 Department of Cardiology, Sint Antonius Hospital, Nieuwegein, The Netherlands; 7 Department of Cardiology, Catharina Hospital, Eindhoven, The Netherlands; 8 Department of Cardiology, Amsterdam UMC, University of Amsterdam, Amsterdam, The Netherlands

**Keywords:** Transcatheter Aortic Valve Implantation, Time Factors, Sex Factors, Mortality, Stroke, Permanent pacemaker implantation

## Abstract

**Background:**

Patients undergoing a transcatheter aortic valve implantation (TAVI) are at risk for Major Adverse Cardiac Events (MACE). We describe temporal trends of TAVI-related MACEs, stratified by age and sex.

**Methods:**

We performed a retrospective analysis of TAVI patients from the Netherlands Heart Registration (NHR) between 2013 and 2022. The outcomes were: mortality at 30 days, mortality at one year, permanent pacemaker implantation at 30 days (PPI), major vascular complication at 30 days (MVC), and stroke at three days. We calculated incidence and trends in TAVI patients and their outcomes.

**Results:**

The cohort consisted of 19,746 TAVI patients, with a mean age of 80 years. The annual number of TAVI procedures increased over the years from 786 to 2876 (*p* < 0.001). Initially, more women received TAVI, but the trend shifted over time to more men (*p* < 0.001). Outcomes incidence was: 30-day mortality (3.3%), one-year mortality (10.6%), PPI (10.7%), MVC (2.9%), and stroke (2.0%). Incidence of both mortality outcomes decreased over time (6.7% to 2.7%, and 15.8% to 8.8% for 30-day and one-year mortality, respectively), as did PPI (12.3% to 10.4%) and MVC (3.6% to 2.5%). Women had a higher incidence of MVC and stroke. Men had a higher incidence of one-year mortality and PPI, and their incidence increased more with age than it did in women.

**Conclusion:**

The volume of TAVI procedures has increased significantly over time, while mortality, PPI, MVC, and MACE incidence have significantly decreased. Sex-specific differences in MACE outcome incidence were present.

**Supplementary Information:**

The online version of this article (10.1007/s12471-025-02006-6) contains supplementary material, which is available to authorized users.

## What’s new?

- Volume of TAVIs in the Netherlands has more than doubled in the last decade
- Since 2017, more men have been receiving a TAVI than women
- Decreasing trends over time in all MACE outcome incidence apart from stroke
- Differences between sexes in case-mix and MACE incidence were present

## Introduction

Transcatheter aortic valve implementation (TAVI) has become the standard treatment for elderly patients with aortic stenosis and increased surgical risk [1, 2]. Over the last years, the number of TAVI procedures has increased as the indication for TAVI expanded from high to intermediate and intermediate-low operative risk. This expanded use of TAVI could have led to a change in adverse outcomes and complications. Adverse outcomes can result in prolonged hospital stays and higher costs. Complications after TAVI have been associated with mortality and with a negative impact on the quality of life [3].

According to the Valve Academic Research Consortium-2 criteria (VARC-2), TAVI-related Major Adverse Cardiac Events (MACE) include mortality, minor vascular complications, major vascular complications (MVC), stroke, renal failure, and aortic regurgitation [3, 4]. In addition to the MACE endpoints, the VARC‑2 includes the need for a permanent pacemaker implantation (PPI) as an outcome reflecting the treatment of conduction disturbances, such as a complete atrioventricular block.

Recently reported rates of TAVI-related 30-day mortality incidence have varied between 3% and 12% [5–7]. Similarly, incidence in 30-day PPI has varied between 6% and 16% [8–12], of MVC between 4% and 6% [5, 13–16], and of stroke between 1% and 7% [2, 17–21]. Despite its relatively low incidence, stroke is a devastating complication and is associated with poor prognosis, and perhaps most importantly in these patients, a low quality of life.

Most TAVI-related patient outcome studies describe outcome rates in the total TAVI population. Specific studies that focused on detailed incidence stratified by sex or different age groups are scarce. MACE data that is more age- and sex-specific would provide better insight for various subgroups of patients. In addition, it would support better individual patient-tailored information and decision-making. Each MACE outcome might also have a different impact on the individual patient, especially after extending TAVI to younger patients and to patients at lower operative risk.

This study has two aims. The first is to describe the incidence and trends of the TAVI-related MACEs in the Netherlands between 2013 and 2022. The second aim is to specifically investigate the incidence of these TAVI-related MACEs among the various age groups and in women versus men.

## Methods

### Study design and population

We conducted a nationwide multicenter retrospective observational study in a national cohort using prospectively entered data from the Netherlands Heart Registration (NHR) [22]. In the Netherlands, 15 heart centres perform TAVI procedures. Data entry to the NHR is mandatory. Thus, the records for all TAVI procedures in the Netherlands are stored in the NHR. These data include demographics, clinical characteristics, intervention risk factors, procedural details, mortality status, complications, and follow-up data after hospital discharge. In the domain of TAVI, the Netherlands has shown to have a slightly conservative adoption, performing fewer TAVI procedures per million people than the average across European countries [23].

For this study, we obtained anonymized data on each performed TAVI procedure from the NHR in the ten-year period between January 1, 2013, and December 31, 2022. For each patient to be included in the current study, a mortality status was required.

### Measured outcomes

We focused on the incidence and trends of the following adverse outcomes that were available in our dataset. These were: 1) early (30–day) mortality, 2) one-year mortality, 3) need for PPI within the first 30–days, 4) occurrence of MVC within the first 30-days, 5) occurrence of stroke within the first 72–hours, and 6) a combined MACE endpoint which is a combination of outcomes 1, 2, 4, and 5 [4].

### Statistical analysis

The full statistical analysis plan is described in the Electronic Supplementary Material, Appendix B, Section Methods.

## Results

The complete NHR TAVI cohort between 2013–2022 consisted of 20,591 patients. Mortality status was unknown in 845 patients (4.1%). Therefore, our study cohort consisted of the remaining 19,746 TAVI patients. A flowchart for the patients’ selection is available in Figure S1. Table S1 shows baseline characteristics of the study population.

**Fig. 1:**
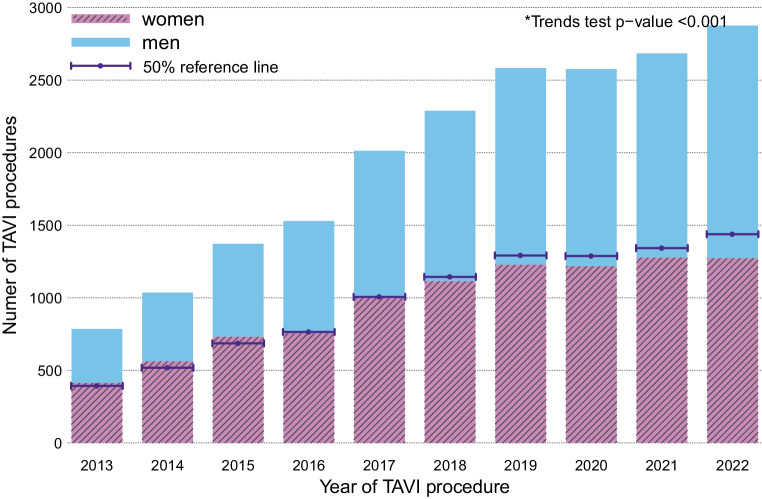
Total number of all TAVI procedures performed in the Netherlands (*N* = 19,746) in each calendar year, stratified by sex. The height of each bar represents the total volume of TAVI procedures performed in the Netherlands for the corresponding year. The pink and blue sections of each bar represent the volumes of women and men, respectively, who received a TAVI. A 50% volume reference line is added at the middle of each bar. *p-values computed using the Cochran Armitage test for trends on the combined (women and men) number of TAVIs per year

The number of TAVI procedures increased significantly over the years from 786 in 2013 to 2876 in 2022 (Fig. 1). The mean age of the TAVI patients was 79.6 years with a standard deviation of 6.7. Compared to men, women were on average 1.5 years older, had mostly better health indicators in the measured variables, except for poor mobility and functional New York Heart Association (NYHA) class (Tab. S1). Over the years, the TAVI patient population has experienced a decrease in their EuroSCORE-II risk score values (median EuroSCORE-II 4 during 2013–2015 versus 2.7 during 2020–2022), as well as a change in procedure modalities (anaesthesia used in 72% of procedures during 2013–2015 versus 33% in 2020–2022, transfemoral access route in 74% of TAVIs during 2013–2015, versus 90% in 2020–2022) (Table S2). During the first three years of the cohort, more women received a TAVI than men. However, the proportion between men and women has slowly been shifting in the direction of more men receiving a TAVI (Fig. 1, Table S4).

**Fig. 2:**
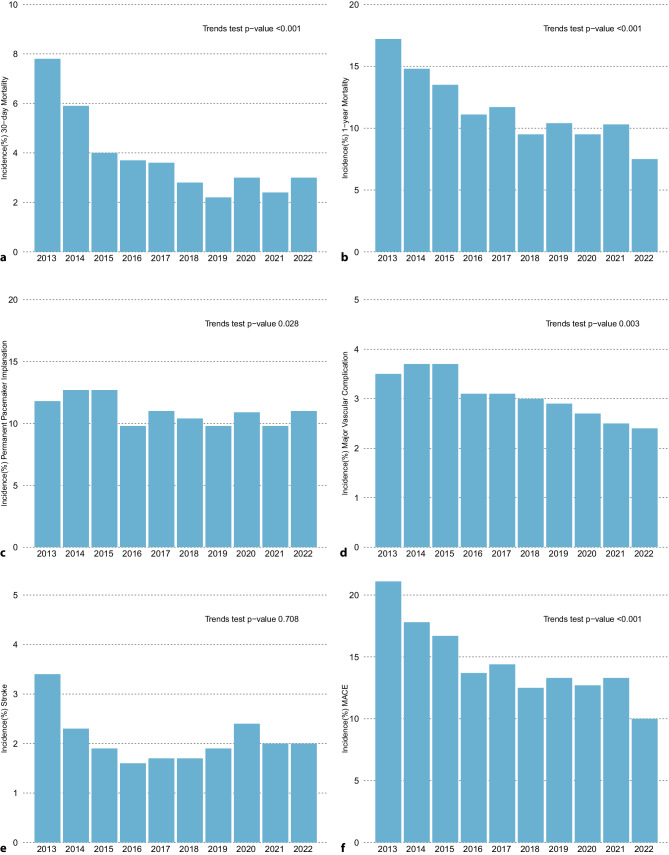
Annual (2013 up to 2022) incidence in TAVI patients in the Netherlands of: **a** 30-day mortality, **b** One-year mortality, **c** Permanent pacemaker implantation, **d** Major vascular complication, and **e** Stroke. *p-values computed using the Cochran-Armitage test for trends. For performing the trend test, calendar years were grouped into two-year periods (2013–2014, 2015–2016, 2017–2018, 2019–2020, 2021–2022)

The incidence of TAVI-related MACEs was as follows: 30-day mortality (3.3%), one-year mortality (10.6%), PPI (10.7%), MVC (2.9%), stroke (2.0%), and combined MACE (13.6%) (Table S1, Table S3). Trends of decreasing incidence rates over the years were observed for all outcomes apart from stroke (Fig. 2, Table S3). The incidence of 30-day mortality significantly decreased over the years from 6.7% in 2013–2014 to 2.7% 2021–2022 (*p* < 0.001). The one-year mortality also declined from 15.8% in 2013–2014 to 8.8% in 2021–2022 (i < 0.001). Incidence in PPI seemed to mostly decrease between the years 2015 to 2016, after which they remained stable (*p* = 0.03). MVC rates have also been decreasing since 2015 onward (*p* = 0.003). Incidence in stroke was decreasing between the years 2013 to 2016.

When stratifying patient outcomes by sex, we found women to have a higher incidence of MVC and stroke, and a lower incidence of PPI and one-year mortality compared to men (Table S1, Figure S2). These differences between sexes have remained so through the years. The difference in PPI rates between women and men has been further increasing in the last two years (2021–2022) (Figure S2). Patients, and especially women, who had a transfemoral TAVI access route were less likely to suffer from a MVC or stroke outcome (Table S5).

Analysis of trends in TAVI MACE with respect to patient age as a continuous variable revealed that the incidence of 30-day and one-year mortality followed a parabolic shape where the incidence of a mortality outcome would first decrease with age in the younger patients, and then later increase with age in the older patients (Figure S3). The incidence of PPI, MVC, and stroke followed a more linear relationship with patient age, where they would increase with age. Analysis by three age groups (< 75, 75–80, > 80 years) showed that for the middle age group (75–80), 30–day and one-year mortality occurred least frequently, while MVC occurred most frequently (Table S6). PPI occurred less often in the youngest age group (< 75).

**Fig. 3:**
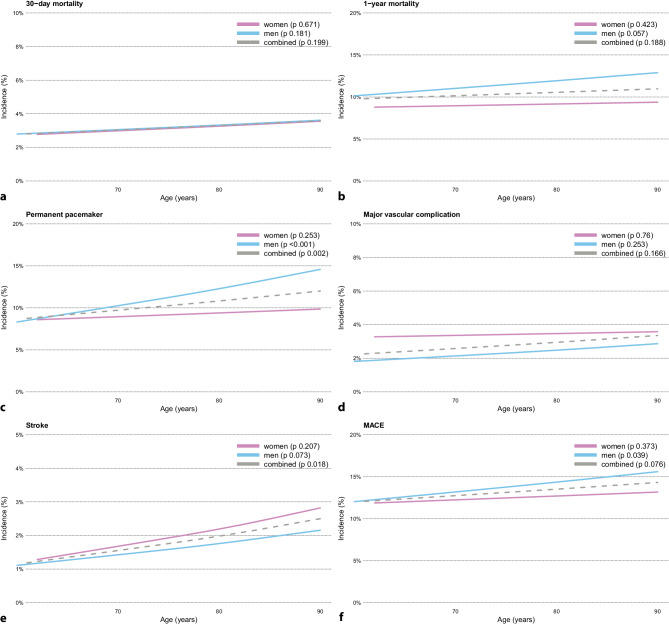
Incidence of each of the TAVI-related patient MACE outcomes per age (in years) for the 19,746 TAVI patients stratified by sex (bottom 1.25 and top 98.725 age percentiles were trimmed). *p-values: significance of using age as a linear predictor in the corresponding outcomes by a logistic regression model

When stratifying by sex, in terms of one-year mortality for women, there was no increase in incidence rates with respect to age. Contrary to this, in men, there was a non-significant increase in one-year mortality incidence with age (Fig. 3). Similarly, for the PPI outcome, there was a significant increase in incidence with age for men when compared to women.

## Discussion

### Main findings

In this national cohort of 19,746 Dutch TAVI patients, there was a strong increase in TAVI procedures and a significant reduction in mortality, PPI, and MVC over the recent decade between 2013 and 2022.

Since 2018, more men have been treated with TAVIs annually compared to women. A potential reason for this shift could be changes in the patient risk profile.

In earlier years, only high-risk patients, i.e., those who did not qualify for Surgical Aortic Valve Replacement (SAVR), were selected for a TAVI. As age is positively related to risk severity, and women tend to have longer lifespans than men, selecting higher risk patients would also mean selecting older patients, and thus more women.

TAVI techniques and outcomes improved; lower-risk patients underwent TAVI. This lower risk usually included younger patients with an accompanying different proportion of men and women receiving TAVIs.

From our analysis of MACE incidence rates between sexes, we found differences for all outcomes with the exception of 30-day mortality. Women had higher rates of MVC and stroke, while men were more at risk of mortality one year after a TAVI, as well as receiving a PPI. These differences in outcome rates between men and women were also shown to hold across the different age groups. The reduction in annual PPI and MVC rates was more pronounced in women than in men (Figure S2).

We also noticed that incidence rates of one-year mortality, and PPI (and potentially for MVC and stroke as well) behaved differently among men and women with respect to age. While age, sex, and other observed factors could help understand the changes in TAVI patient outcomes, factors such as device evolution, refinements in procedural techniques, or other unexamined influences may have contributed to these observed changes as well.

Our findings on TAVI-related MACE incidence rates support other published studies. Mortality rates were comparable to other studies [5–7], as were PPI rates [8–12, 24]. The MVC incidence (2.9%) was lower in the current cohort when compared to previous articles (4% to 9.3%) [2, 5, 13–16, 25]. Stroke rate in this cohort was 2%, which is comparable to other reports (0.6% to 6.9%) [2, 17–21].

Few studies have investigated differences between men and women who underwent a TAVI [26–28].

This study adds to the existing literature by offering insights into the incidence trends of TAVI-related patient MACEs in different age groups, as well as differences between men and women.

### Literature comparison

Ravani et al. reported that the ratio between men and women who received a TAVI had mostly remained equal [26]. This is in contrast to our study findings, suggesting that TAVI patient selection criteria could vary between countries.

The multicenter study by Itzhaki Ben Zadok et al. found their TAVI cohort to comprise more women than men, however, this difference was less pronounced in the later time period of the cohort [27]. Although limited, this provides some supporting evidence that the trend of increasing proportion of men observed for Dutch TAVI patients could be occurring in other countries as well.

Bleiziffer et al. looked at procedure volume data for Surgical Aortic Valve Replacement (SAVR) and TAVIs performed across 12 countries in Europe [28]. They reported that in 2020, more Aortic Valve Replacements (AVR) (i.e., either TAVI or SAVR) were done on men than women. Adjusting patient selection in AVR can have a trickle-down effect in both TAVI and SAVR patient composition. From the patients who received an AVR, women received a TAVI more often than men [28]. This suggests that our observed trend of more men receiving TAVI than women could at least partially be driven by a change in the proportion of men being elected for AVR. That is, if more men get elected for AVR, then more men will also receive a TAVI (assuming all else is equal). Looking into the reports of AVR volumes in the Netherlands, we see that AVR volumes have been increasing [23].

A more comprehensive discussion of our results in the light of previous studies is described in Electronic Supplementary Material, Appendix B, Section Discussion.

### Strengths and limitations

Our study has the inherent limitations of any retrospective registry analysis. To obtain reliable data, the NHR has an advanced, certified data quality control system in place to ensure completeness and quality of data [29].

From the five available patient outcomes, mortality had a one-year and 30-day follow-up available, whilst PPI and MVC only had 30-day follow-up, and stroke only had a three-day follow-up. As a result, the reported combined MACE incidence likely underestimates the true one-year rate. Heterogeneity in endpoint definitions between this study and previous reports could account for differences in MVC and stroke rates.

Another limitation of the current study is related to the types of TAVI prostheses used. Since we included TAVI patients starting from 2013, different TAVI prosthesis types (self-expanding and balloon-expanding) and different generations (old and new) were used. However, this heterogeneous cohort of patients might reflect the current clinical practice. Unfortunately, our national NHR dataset does not capture details on prostheses type.

Strengths of the current study include its use of one of the largest cohorts investigating the incidence and trends of TAVI-related patient MACEs, with in-depth analyses among different age and sex categories. This study uses a national registry cohort with excellent completeness of registration.

### Implications and future studies

The occurrence of TAVI-related MACE mandates ongoing critical evaluation of risk factors associated with these adverse outcomes. Such continuous research utilizing data about the used TAVI prosthesis devices, or procedure access route, can contribute to the quality improvement and gaining of insights into the success of the centres with low MACE rates. This type of research allows physicians to evaluate TAVI-related MACE and address them with the aim to effectively improving clinical outcomes. Clinicians can use the observed sex-specific trends to tailor pre-procedural counselling by highlighting the higher risks of MVC and stroke in women (especially for non-transfemoral access route TAVIs), and the increased likelihood of PPI and one-year mortality in men. Post-TAVI management strategies could also be adjusted, with closer monitoring for vascular complications and stroke in women, and targeted interventions to reduce conduction disturbances and optimize long-term outcomes in men, particularly in higher-risk groups.

In order to evaluate and improve clinical outcomes, better identification of risk factors needs to be included in large registries. It will allow the development of outcome-related risk prediction models for TAVI patients, which are highly needed for individualized and patient-tailored information and treatment. The current NHR-provided TAVI variables set has limited capability for the development of such prediction models for separate MACE outcomes besides mortality. As such, for better prediction of PPI, we would suggest adding relevant echocardiographic characteristics (left ventricular end-diastolic and end-systolic diameters, interventricular septum thickness, aortic valve annulus diameter), electrocardiographic characteristics (PR-interval, degree of atrioventricular block, QRS-duration and axis, and the presence of right or left bundle branch block), as well as patient frailty scores. Future efforts at developing outcome-specific risk prediction models for TAVI patients should further investigate the interaction between sex and age as predictors. As patient case-mix and TAVI-related outcomes can vary between different hospitals, an additional area of future research could address TAVI trends over different centers, or types of centers, such as high-volume versus low-volume centers, and academic versus non-academic centers.

## Conclusion

The number of performed TAVI procedures has increased between 2013 and 2022. While initially more women received TAVI, the trend shifted over time to more men.

Mortality, PPI, MVC, and MACE incidence have significantly decreased over the years. Decreases in PPI and MVC rates were more pronounced in women than in men. With respect to older age, increases in one-year mortality and PPM incidence were greater for men than for women. Men also had a higher overall incidence of one-year mortality and PPI compared to women. Women had a higher incidence of MVC, and stroke compared to men.

## Supplementary Information

List of members of the Registration Committees of the Netherlands Heart Registration

Comprised of:
- Appendix A (supplementary figures and supplementary tables)
- Appendix B (supplementary methods and supplementary discussion)
