# Supplementary material for: Incidence and trends of patient MACE outcomes after Transcatheter Aortic Valve Implantation (TAVI): analysis by age and sex
Source: Neth Heart J. 2025 Dec 17;34(1):36–44. doi: 10.1007/s12471-025-02006-6 (PMC12779812; doi:10.1007/s12471-025-02006-6)
Supplement: Supplementary file 1 — List of members of the Registration Committees of the Netherlands Heart Registration [file 12471_2025_2006_MOESM1_ESM.docx]

**[List of members of the Registration Committees of the Netherlands Heart Registration](https://static-content.springer.com/esm/art%3A10.1007%2Fs12471-024-01877-5/MediaObjects/12471_2024_1877_MOESM5_ESM.docx)**

The following physicians are members of **the NHR THI Registration Committee**. They represent the hospitals that have provided the data for this study. Contact with the NHR THI Registration Committee can be via the e-mail info@nederlandsehartregistratie.nl

| **Name** | **Center** |
| --- | --- |
| Dr. B.J.L. van den Branden | Amphia Ziekenhuis |
| Dr. R. Delewi | Amsterdam UMC |
| Dr. W.A.L. Tonino | Catharina Ziekenhuis |
| Prof. dr. N.M.D.A. van Mieghem | Erasmus Medisch Centrum |
| Dhr. C.E. Schotborgh | HagaZiekenhuis |
| Dr. R.S. Hermanides | Isala |
| Dhr. F. van der Kley | Leids Universitair Medisch Centrum |
| Dr. P. Vriesendorp | Maastricht UMC+ |
| Dhr. F. Porta | Medisch Centrum Leeuwarden |
| Dhr. K.G. van Houwelingen | Medisch Spectrum Twente |
| Dr. G. Amoroso | Onze Lieve Vrouwe Gasthuis |
| Mevr. M. van Wely | Radboudumc |
| Dr. L. Timmers | St. Antonius Ziekenhuis |
| Dr. M. Voskuil | UMC Utrecht |
| Dhr. H.W. van der Werf | Universitair Medisch Centrum Groningen |
