# Supplementary material for: Incidence and trends of patient MACE outcomes after Transcatheter Aortic Valve Implantation (TAVI): analysis by age and sex
Source: Neth Heart J. 2025 Dec 17;34(1):36–44. doi: 10.1007/s12471-025-02006-6 (PMC12779812; doi:10.1007/s12471-025-02006-6)
Supplement: Supplementary file 2 — Comprised of: - Appendix A (supplementary figures and supplementary tables) - Appendix B (supplementary methods and supplementary discussion) [file 12471_2025_2006_MOESM2_ESM.docx]

Incidence and trends of patient MACE outcomes after Transcatheter Aortic Valve Implantation (TAVI): analysis by age and sex

Contents

[Appendix A. 2](#_Toc203755377)

[Supplementary figure and table captions 2](#_Toc203755378)

[Supplementary tables 3](#_Toc203755379)

[Supplementary figures 8](#_Toc203755380)

[Appendix B 11](#_Toc203755381)

[Supplementary Methods 11](#_Toc203755382)

[Statistical analysis 11](#_Toc203755383)

[Supplementary Discussion 12](#_Toc203755384)

# Appendix A.

## Supplementary figure and table captions

E-Table 1. Baseline characteristics of the total TAVI study population, and stratified by sex.

E-Table 2. Summary of TAVI patient characteristics and procedure-specific variables of the total TAVI study population grouped by year of the procedure (2013-2015, 2016-2019, and 2020-2022).

E-Table 3. Incidence of patient MACE outcomes after TAVI procedures and the trends over the years (2013-2022) for the 19,746 TAVI-patients from the Netherlands Heart Registration registry (NHR).

E-Table 4. Number of TAVI procedures performed in the Netherlands per calendar year stratified by sex (N= 19,746).

E-Table 5. Incidence of Major Vascular Complication (MVC) within 30-days (A), and Stroke within 3-days (B), after TAVI of the total study population across five two-year periods (2013-2014, 2015-2016, 2017-2018, 2019-2020, and 2021-2022), grouped by sex and TAVI procedure access route (transfemoral versus non-transfemoral).

E-Table 6. Incidence and numbers of MACE outcomes in different age groups of the 19,746 TAVI patients. The patients are sub-grouped according to the age groups <75, 75-80, and >80 year.

E-Figure 1. Flowchart for TAVI-patients included in the study.

E-Figure 2. Annual (2013 up to 2022) MACE incidence in TAVI patients in the Netherlands stratified by sex. A. 30-day mortality, B. One-year mortality, C. Permanent pacemaker implantation, D. Major vascular complication, E. Stroke, and F. MACE.

E-Figure 3. Incidence of each of the TAVI-related MACE outcomes per age (in years) for the 19,746 TAVI-patients (bottom 1.25 and top age 98.725 percentiles were trimmed).

## Supplementary tables

E-Table 1. Baseline characteristics of the total TAVI study population, and stratified by sex.

| **Variable** | **Total**  **Population** | |  | **Women** | | **Men** | |  |
| --- | --- | --- | --- | --- | --- | --- | --- | --- |
|  | **N=19,746** | **Missing %** |  | **N=9,576** | **Missing %** | **N=10,170** | **Missing %** | **p-value** |
| ***Continuous variables*** |  |  |  |  |  |  |  |  |
| **Age (years) (mean (SD))** | 79.6 (6.74) | 0% |  | 80.4 (6.51) | 0% | 78.9 (6.88) | 0% | <0.001 |
| **BSA (mean (SD))** | 1.9 (0.22) | 2% |  | 1.8 (0.2) | 2.1% | 2 (0.19) | 2% | <0.001 |
| **Body Mass Index BMI (kg/m^2^) (median (IQR))** | 26.4 (24;30) | 2% |  | 26.6 (24;30) | 2.1% | 26.3 (24;29) | 2% | 0.245 |
| **LVEF (median (IQR))** | 55 (40;55) | 2.3% |  | 55 (50;55) | 2.1% | 55 (40;55) | 2.4% | 0.221 |
| **Serum creatinine (μmol/L) (median (IQR))** | 92 (75;115) | 0.4% |  | 81 (67;100) | 0.4% | 102 (85;127) | 0.4% | 0.245 |
| **eGFR (mean (SD))** | 59.1 (19.46) | 0.4% |  | 59 (19) | 0.4% | 59.3 (19.89) | 0.4% | 0.359 |
| **Pulmonary artery systolic pressure (median (IQR))** | 25 (25;32) | 13.1% |  | 25 (25;33) | 13.1% | 25 (25;30) | 13.2% | 0.221 |
| **EuroSCORE-II (median (IQR))** | 3 (2;5) | 33.2% |  | 3 (2;5) | 32.8% | 2.9 (2;5) | 33.6% | 0.245 |
| ***Outcomes*** |  |  |  |  |  |  |  |  |
| **Early (30-days) mortality (%)** | 657 (3.3) | 0% |  | 321 (3.4) | 0% | 336 (3.3) | 0% | 0.881 |
| **One-year mortality (%)** | 2100 (10.6) | 0% |  | 886 (9.3) | 0% | 1214 (11.9) | 0% | <0.001 |
| **Permanent pacemaker implantation (%)** | 2043 (10.7) | 3.6% |  | 862 (9.3) | 3.5% | 1181 (12.1) | 3.7% | <0.001 |
| **Major vascular complication (%)** | 494 (2.9) | 14.4% |  | 283 (3.4) | 13.6% | 211 (2.4) | 15.1% | <0.001 |
| **Stroke (%)** | 381 (2) | 3.7% |  | 207 (2.2) | 3.4% | 174 (1.8) | 3.9% | 0.024 |
| **MACE (%)** | 2682 (13.6) | 0% |  | 1221 (12.8) | 0% | 1461 (14.4) | 0% | 0.001 |
| ***Binary categorical variables*** |  |  |  |  |  |  |  |  |
| **Female Gender (%)** | 9576 (48.5) | 0% |  | 9576 (100) | 0% | 10170 (100) | 0% | NA |
| **Chronic lung disease (%)** | 3750 (19.1) | 0.3% |  | 1588 (16.6) | 0.3% | 2162 (21.3) | 0.3% | <0.001 |
| **Extra-cardiac arteriopathy (%)** | 3673 (18.7) | 0.7% |  | 1366 (14.4) | 0.7% | 2307 (22.9) | 0.8% | <0.001 |
| **Neurological dysfunction (%)** | 694 (3.8) | 7.4% |  | 301 (3.4) | 7.8% | 393 (4.2) | 6.9% | 0.007 |
| **Previous cardiac surgery (%)** | 3658 (18.8) | 1.6% |  | 1079 (11.4) | 1.5% | 2579 (25.8) | 1.6% | <0.001 |
| **Critical preoperative state (%)** | 101 (0.5) | 0.7% |  | 42 (0.4) | 0.6% | 59 (0.6) | 0.7% | 0.196 |
| **Unstable angina (%)** | 66 (0.3) | 1.1% |  | 25 (0.3) | 1.2% | 41 (0.4) | 1.1% | 0.108 |
| **Recent MI (%)** | 368 (1.9) | 0.8% |  | 158 (1.7) | 0.7% | 210 (2.1) | 0.9% | 0.036 |
| **Dialysis (%)** | 198 (1) | 1.2% |  | 63 (0.7) | 1.3% | 135 (1.3) | 1.1% | <0.001 |
| **Poor mobility (%)** | 1679 (10) | 14.8% |  | 928 (11.5) | 15.8% | 751 (8.6) | 13.8% | <0.001 |
| **CCS class IV angina (%)** | 374 (2.1) | 10.6% |  | 164 (1.9) | 11.4% | 210 (2.3) | 9.8% | 0.078 |
| **Procedure weight (2 operations) (%)** | 3 (0.02) | 2.3% |  | 1 (0.01) | 2.5% | 2 (0.02) | 2.1% | 1 |
| **Previous CVA (%)** | 2048 (10.4) | 0.7% |  | 889 (9.3) | 0.6% | 1159 (11.5) | 0.7% | <0.001 |
| **Previous aortic valve surgery (%)** | 901 (4.7) | 2.3% |  | 419 (4.5) | 2.4% | 482 (4.8) | 2.2% | 0.234 |
| **Previous permanent pacemaker (%)** | 1556 (8.2) | 3.7% |  | 616 (6.7) | 4.1% | 940 (9.6%) | 3.3% | <0.001 |
| **Anesthesia (%)** | 9357 (48.4) | 2% |  | 4470 (47.7) | 2.1% | 4887 (49) | 1.9% | 0.055 |
| **Balloon pre-TAVI (%)** | 8242 (44) | 5.1% |  | 3933 (43.5) | 5.6% | 4309 (44.5) | 4.7% | 0.067 |
| ***Non-binary categorical variables*** |  |  |  |  |  |  |  |  |
| **Functional NYHA class** |  | 6.6% |  |  | 6.9% |  | 6.3% |  |
| NYHA class I (%) | 1833 (9.9) |  |  | 779 (8.7) |  | 1054 (11.1) |  | <0.001 |
| NYHA class II (%) | 5948 (32.3) |  |  | 2774 (31.1) |  | 3174 (33.3) |  | <0.001 |
| NYHA class III (%) | 9434 (51.2) |  |  | 4757 (53.4) |  | 4677 (49.1) |  | <0.001 |
| NYHA class IV (%) | 1222 (6.6) |  |  | 602 (6.8) |  | 620 (6.5) |  | 0.6 |
| **Diabetes Mellitus (DM)** |  | 1.3% |  |  | 1.4% |  | 1.2% |  |
| No DM (%) | 14237 (73.1) |  |  | 7017 (74.3) |  | 7220 (71.9) |  | <0.001 |
| DM not on medication (%) | 1062 (5.4) |  |  | 556 (5.9) |  | 506 (5) |  | 0.011 |
| DM on medication (%) | 4190 (21.5) |  |  | 1871 (19.8) |  | 2319 (23.1) |  | <0.001 |
| **Access route** |  | 0.9% |  |  | 0.9% |  | 0.9% |  |
| Access route Transfemoral (%) | 16486 (84.2) |  |  | 8097 (85.3) |  | 8389 (83.2) |  | <0.001 |
| Access route subclavian artery (%) | 1040 (5.3) |  |  | 479 (5) |  | 561 (5.6) |  | 0.113 |
| Access route Transapical (%) | 905 (4.6) |  |  | 380 (4) |  | 525 (5.2) |  | <0.001 |
| Access route direct aortic (%) | 1141 (5.8) |  |  | 538 (5.7) |  | 603 (6) |  | 0.365 |
| **Procedure acuity** |  | 1.4% |  |  | 1.4% |  | 1.4% |  |
| Elective (%) | 17331 (89) |  |  | 8480 (89.8) |  | 8851 (88.2) |  | 0.001 |
| Urgent (%) | 2086 (10.7) |  |  | 944 (10) |  | 1142 (11.4) |  | 0.002 |
| Emergency (%) | 54 (0.3) |  |  | 16 (0.2) |  | 38 (0.4) |  | 0.008 |

For continuous variables, values are mean and standard deviation (SD), or median and intra-quartile range (IQR) where appropriate.
For binary and categorical variables, values are number (n) and percentage (%).

Abbreviations: BMI = Body mass index; Balloon pre-TAVI = Balloon aortic valvuloplasty prior to date of TAVI; CCS class = Canadian Cardiovascular Society grading of angina pectoris; CKD = Chronic Kidney Disease; CVA = cerebrovascular accident; DM = Diabetes mellitus; eGFR = estimated Glomerular Filtration Rate; LVEF = Left Ventricular Ejection Fraction; MI = myocardial infarction; NYHA = New York Heart Association functional Classification; PABV = Percutaneous Aortic Balloon Valvuloplasty (TAVI post-dilation), Post-MI VSR = poet myocardial infarction ventricular septal rupture; SBA = surface body area; sPAP = systolic Pulmonary Arterial Pressure.

E-Table 2. Summary of TAVI patient characteristics and procedure-specific variables of the total TAVI study population grouped by year of the procedure (2013-2015, 2016-2019, and 2020-2022).

| **Year of procedure** | **2013-2015** | **2016-2019** | **2020-2022** |
| --- | --- | --- | --- |
| **Number of TAVIs performed** | **N=3,194** | **N=8,415** | **N=8,137** |
| **Variable** |  |  |  |
| ***Patient characteristics*** |  |  |  |
| **Age (years) (mean (SD))** | 80 (7.22) | 79.6 (6.74) | 79.5 (6.53) |
| **EuroSCORE-II (median (IQR))** | 4 (3;7) | 3 (2;5) | 2.7 (2;5) |
| **Female Gender (%)** | 1709 (53.5%) | 4097 (48.7%) | 3770 (46.3%) |
| **NYHA class I (%)** | 233 (8.4%) | 905 (11.8%) | 695 (8.7%) |
| **NYHA class II (%)** | 629 (22.8%) | 2218 (28.8%) | 3101 (38.8%) |
| **NYHA class III (%)** | 1647 (59.6%) | 4110 (53.5%) | 3677 (46%) |
| **NYHA class IV (%)** | 254 (9.2%) | 456 (5.9%) | 512 (6.4%) |
| **Diabetes Mellitus (%)** | 888(27.8%) | 2283(27.1%) | 2141(26.3%) |
| **Poor mobility (%)** | 121 (8.8%) | 693 (9.3%) | 865 (10.8%) |
| **CCS class IV angina (%)** | 47 (2.6%) | 214 (2.7%) | 113 (1.4%) |
| **Previous cardiac surgery (%)** | 666 (22.5%) | 1658 (19.8%) | 1334 (16.5%) |
| **Previous permanent pacemaker (%)** | 212 (8%) | 688 (8.3%) | 656 (8.1%) |
| **Chronic lung disease (%)** | 744 (23.5%) | 1640 (19.5%) | 1366 (16.8%) |
| **Extra-cardiac arteriopathy (%)** | 805 (25.5%) | 1696 (20.2%) | 1172 (14.6%) |
| ***Procedural variables*** |  |  |  |
| **Anesthesia (%)** | 2194 (71.7%) | 4496 (55%) | 2667 (32.9%) |
| **Access route Transfemoral (%)** | 2342 (73.9%) | 6907 (83.1%) | 7237 (89.5%) |
| **Balloon pre-TAVI (%)** | 1738 (68.8%) | 3188 (39.2%) | 3316 (41.1%) |
| **Procedure weight (2 operations) (%)** | 28 (1%) | 3 (0.04%) | 140 (1.7%) |

E-Table 3. Incidence of patient MACE outcomes after TAVI procedures and the trends over the years (2013-2022) for the 19,746 TAVI-patients from the Netherlands Heart Registration registry (NHR).

| **TAVI patient outcomes** | **Events and  total cases**  **2013-2022** | **Incidence  %** |  | **Trends over the years** | | | | | | | | | |  |  |
| --- | --- | --- | --- | --- | --- | --- | --- | --- | --- | --- | --- | --- | --- | --- | --- |
|  |  |  |  | **2013-2014** | **%** | **2015-2016** | **%** | **2017-2018** | **%** | **2019-2020** | **%** | **2021-2022** | **%** |  | **Trend test**  **p-value**† |
| **30-day mortality** | 657/19746 | 3.3 |  | 122/1822 | 6.7 | 112/2901 | 3.9 | 136/4302 | 3.2 | 135/5160 | 2.6 | 152/5561 | 2.7 |  | <0.001 |
| **One-year mortality** | 2100/19746 | 10.6 |  | 288/1822 | 15.8 | 354/2901 | 12.2 | 452/4302 | 10.5 | 514/5160 | 10.0 | 492/5561 | 8.8 |  | <0.001 |
| **Permanent pacemaker implantation** | 2043/19037* | 10.7 |  | 215/1748 | 12.3 | 314/2814 | 11.2 | 445/4176 | 10.7 | 515/4970 | 10.4 | 554/5329 | 10.4 |  | 0.028 |
| **Major vascular complication** | 494/16903* | 2.9 |  | 58/1601 | 3.6 | 84/2485 | 3.4 | 114/3726 | 3.1 | 123/4412 | 2.8 | 115/4679 | 2.5 |  | 0.003 |
| **Stroke** | 381/19023* | 2.0 |  | 45/1619 | 2.8 | 45/2604 | 1.7 | 69/4117 | 1.7 | 110/5128 | 2.1 | 112/5555 | 2.0 |  | 0.708 |
| **MACE** | 2682/19746 | 13.6 |  | 350/1822 | 19.2 | 438/2901 | 15.1 | 576/4302 | 13.4 | 671/5160 | 13.0 | 647/5561 | 11.6 |  | <0.001 |

***** From the total 19,746 included patients not all had a known outcome for Permanent pacemaker implantation (PPI), Major vascular complication (MVC), or stroke.
For PPI, 709 records were missing the outcome, hence the total count of records was 19,037;
For MVC, 2843 records were missing the outcome, hence the total count of records was 16,903;
For stroke, 723 records were missing the outcome, hence the total count of records was 19,023;
See E-Supplementary fig. 1 for more information.
† P-values computed using the Cochran Armitage test for trends.

E-Table 4. Number of TAVI procedures performed in the Netherlands per calendar year stratified by sex (N= 19,746).

| **Year of TAVI** | **Women** | **Men** | **Trend P-value*** |
| --- | --- | --- | --- |
|  | (count) | |  |
|  |  |  | <0.001 |
| 2013 | 414 | 372 |  |
| 2014 | 563 | 473 |  |
| 2015 | 732 | 640 |  |
| 2016 | 756 | 773 |  |
| 2017 | 998 | 1015 |  |
| 2018 | 1115 | 1174 |  |
| 2019 | 1228 | 1356 |  |
| 2020 | 1218 | 1358 |  |
| 2021 | 1278 | 1407 |  |
| 2022 | 1274 | 1602 |  |

*p-values computed using the Cochran Armitage test for trends.

E-Table 5. Incidence of Major Vascular Complication (MVC) within 30-days (**A**), and Stroke within 3-days (**B**), after TAVI of the total study population across five two-year periods (2013-2014, 2015-2016, 2017-2018, 2019-2020, and 2021-2022), grouped by sex and TAVI procedure access route (transfemoral versus non-transfemoral).

| **A.** | \| **Major Vascular Complication incidence (%)** \| **Year of TAVI procedure** \| \| \| \| \|  \| \| --- \| --- \| --- \| --- \| --- \| --- \| --- \| \| **2013-2014** \| **2015-2016** \| **2017-2018** \| **2019-2020** \| **2021-2022** \| **Total** \| \| **Combined (women and men)** \|  \|  \|  \|  \|  \|  \| \| Access route Transfemoral \| 37/1313 (2.8%) \| 61/2212 (2.8%) \| 96/3549 (2.7%) \| 91/4348 (2.1%) \| 95/5064 (1.9%) \| 380/16486 (2.3%) \| \| Access route non-Transfemoral \| 21/502 (4.2%) \| 23/653 (3.5%) \| 17/695 (2.4%) \| 32/779 (4.1%) \| 20/484 (4.1%) \| 113/3113 (3.6%) \| \| **Women** \|  \|  \|  \|  \|  \|  \| \| Access route Transfemoral \| 21/723 (2.9%) \| 46/1155 (4.0%) \| 59/1782 (3.3%) \| 50/2099 (2.4%) \| 45/2338 (1.9%) \| 221/8097 (2.7%) \| \| Access route non-Transfemoral \| 10/250 (4.0%) \| 12/319 (3.8%) \| 8/305 (2.6%) \| 18/331 (5.4%) \| 13/206 (6.3%) \| 61/1411 (4.3%) \| \| **Men** \|  \|  \|  \|  \|  \|  \| \| Access route Transfemoral \| 16/590 (2.7%) \| 15/1057 (1.4%) \| 37/1767 (2.1%) \| 41/2249 (1.8%) \| 50/2726 (1.8%) \| 159/8389 (1.9%) \| \| Access route non-Transfemoral \| 11/252 (4.4%) \| 11/334 (3.3%) \| 9/390 (2.3%) \| 14/448 (3.1%) \| 7/278 (2.5%) \| 52/1702 (3.1%) \| |
| --- | --- | --- | --- | --- | --- | --- | --- | --- | --- | --- | --- | --- | --- | --- | --- | --- | --- | --- | --- | --- | --- | --- | --- | --- | --- | --- | --- | --- | --- | --- | --- | --- | --- | --- | --- | --- | --- | --- | --- | --- | --- | --- | --- | --- | --- | --- | --- | --- | --- | --- | --- | --- | --- | --- | --- | --- | --- | --- | --- | --- | --- | --- | --- | --- | --- | --- | --- | --- | --- | --- | --- | --- | --- | --- | --- | --- | --- |

| B. | \| **Stroke incidence (%)** \| **Year of TAVI procedure** \| \| \| \| \|  \| \| --- \| --- \| --- \| --- \| --- \| --- \| --- \| \| **2013-2014** \| **2015-2016** \| **2017-2018** \| **2019-2020** \| **2021-2022** \| **Total** \| \| **ALL (women and men)** \|  \|  \|  \|  \|  \|  \| \| Access route Transfemoral \| 30/1313 (2.3%) \| 33/2212 (1.5%) \| 50/3549 (1.4%) \| 72/4348 (1.7%) \| 97/5064 (1.9%) \| 282/16486 (1.7%) \| \| Access route non-Transfemoral \| 15/502 (3.0%) \| 12/653 (1.8%) \| 17/695 (2.4%) \| 37/779 (4.7%) \| 15/484 (3.1%) \| 96/3113 (3.1%) \| \| **Women** \|  \|  \|  \|  \|  \|  \| \| Access route Transfemoral \| 19/723 (2.6%) \| 19/1155 (1.6%) \| 22/1782 (1.2%) \| 36/2099 (1.7%) \| 52/2338 (2.2%) \| 148/8097 (1.8%) \| \| Access route non-Transfemoral \| 10/250 (4.0%) \| 8/319 (2.5%) \| 12/305 (3.9%) \| 19/331 (5.7%) \| 8/206 (3.9%) \| 57/1411 (4.0%) \| \| **Men** \|  \|  \|  \|  \|  \|  \| \| Access route Transfemoral \| 11/590 (1.9%) \| 14/1057 (1.3%) \| 28/1767 (1.6%) \| 36/2249 (1.6%) \| 45/2726 (1.7%) \| 134/8389 (1.6%) \| \| Access route non-Transfemoral \| 5/252 (2.0%) \| 4/334 (1.2%) \| 5/390 (1.3%) \| 18/448 (4.0%) \| 7/278 (2.5%) \| 39/1702 (2.3%) \| |
| --- | --- | --- | --- | --- | --- | --- | --- | --- | --- | --- | --- | --- | --- | --- | --- | --- | --- | --- | --- | --- | --- | --- | --- | --- | --- | --- | --- | --- | --- | --- | --- | --- | --- | --- | --- | --- | --- | --- | --- | --- | --- | --- | --- | --- | --- | --- | --- | --- | --- | --- | --- | --- | --- | --- | --- | --- | --- | --- | --- | --- | --- | --- | --- | --- | --- | --- | --- | --- | --- | --- | --- | --- | --- | --- | --- | --- | --- |

E-Table 6. Incidence and numbers of MACE outcomes in different age groups of the 19,746 TAVI patients. The patients are sub-grouped according to the age groups <75, 75-80, and >80 year.

|  | **Age groups of the TAVI patients** | | | | | |  |  |  |
| --- | --- | --- | --- | --- | --- | --- | --- | --- | --- |
| **Outcome** | **<75 years** | **%** | **75-80 years** | **%** | **>80 years** | **%** |  | **Total** | **%** |
| **30-day mortality** | 127 |  | 171 |  | 359 |  |  | 657 |  |
| Number of patients in this group | 3878 | 19.6 | 5737 | 29.1 | 10131 | 51.3 |  | 19746 | 100 |
| Incidence % | 127/3878 | 3.3 | 171/5737 | 3 | 359/10131 | 3.5 |  | 657/19746 | 3.3 |
| p-value^*^ |  | 0.839 |  | 0.082 |  | 0.082 |  |  | 0.161 |
| **1-year mortality** | 434 |  | 540 |  | 1126 |  |  | 2100 |  |
| Number of patients in this group | 3878 | 19.6 | 5737 | 29.1 | 10131 | 51.3 |  | 19746 | 100 |
| Incidence % | 434/3878 | 11.2 | 540/5737 | 9.4 | 1126/10131 | 11.1 |  | 2100/19746 | 10.6 |
| p-value^*^ |  | 0.21 |  | <0.001 |  | 0.025 |  |  | 0.002 |
| **Permanent pacemaker implantation (PPI)** | 366 |  | 607 |  | 1070 |  |  | 2043 |  |
| Number of patients in this group | 3740 | 19.6 | 5538 | 29.1 | 9759 | 51.3 |  | 19037 | 100 |
| Incidence % | 366/3740 | 9.8 | 607/5538 | 11 | 1070/9759 | 11 |  | 2043/19037 | 10.7 |
| p-value^*^ |  | 0.037 |  | 0.513 |  | 0.288 |  |  | 0.114 |
| **Major vascular complication (MVC)** | 79 |  | 158 |  | 257 |  |  | 494 |  |
| Number of patients in this group | 3209 | 19 | 4910 | 29 | 8784 | 52 |  | 16903 | 100 |
| Incidence % | 79/3209 | 2.5 | 158/4910 | 3.2 | 257/8784 | 2.9 |  | 494/16903 | 2.9 |
| p-value^*^ |  | 0.085 |  | 0.145 |  | 0.979 |  |  | 0.141 |
| **Stroke** | 62 |  | 95 |  | 224 |  |  | 381 |  |
| Number of patients in this group | 3700 | 19.5 | 5539 | 29.1 | 9784 | 51.4 |  | 19023 | 100 |
| Incidence % | 62/3700 | 1.7 | 95/5539 | 1.7 | 224/9784 | 2.3 |  | 381/19023 | 2 |
| p-value^*^ |  | 0.113 |  | 0.069 |  | 0.004 |  |  | 0.015 |
| **MACE** | 531 |  | 721 |  | 1430 |  |  | 2682 |  |
| Number of patients in this group | 3878 | 19.6 | 5737 | 29.1 | 10131 | 51.3 |  | 19746 | 100 |
| Incidence % | 531/3878 | 13.7 | 721/5737 | 12.6 | 1430/10131 | 14.1 |  | 2682/19746 | 13.6 |
| p-value^*^ |  | 0.823 |  | 0.008 |  | 0.025 |  |  | 0.023 |

*p-values computed using chi-square testing of the age groups with the corresponding outcome. p-values were calculated both for each age group separately (shown as the p-values next to each age group), and for all three age groups combined (shown as the p-values to the right of the “Total” column).

## Supplementary figures

E-Figure 1. Flowchart for TAVI-patients included in the study.


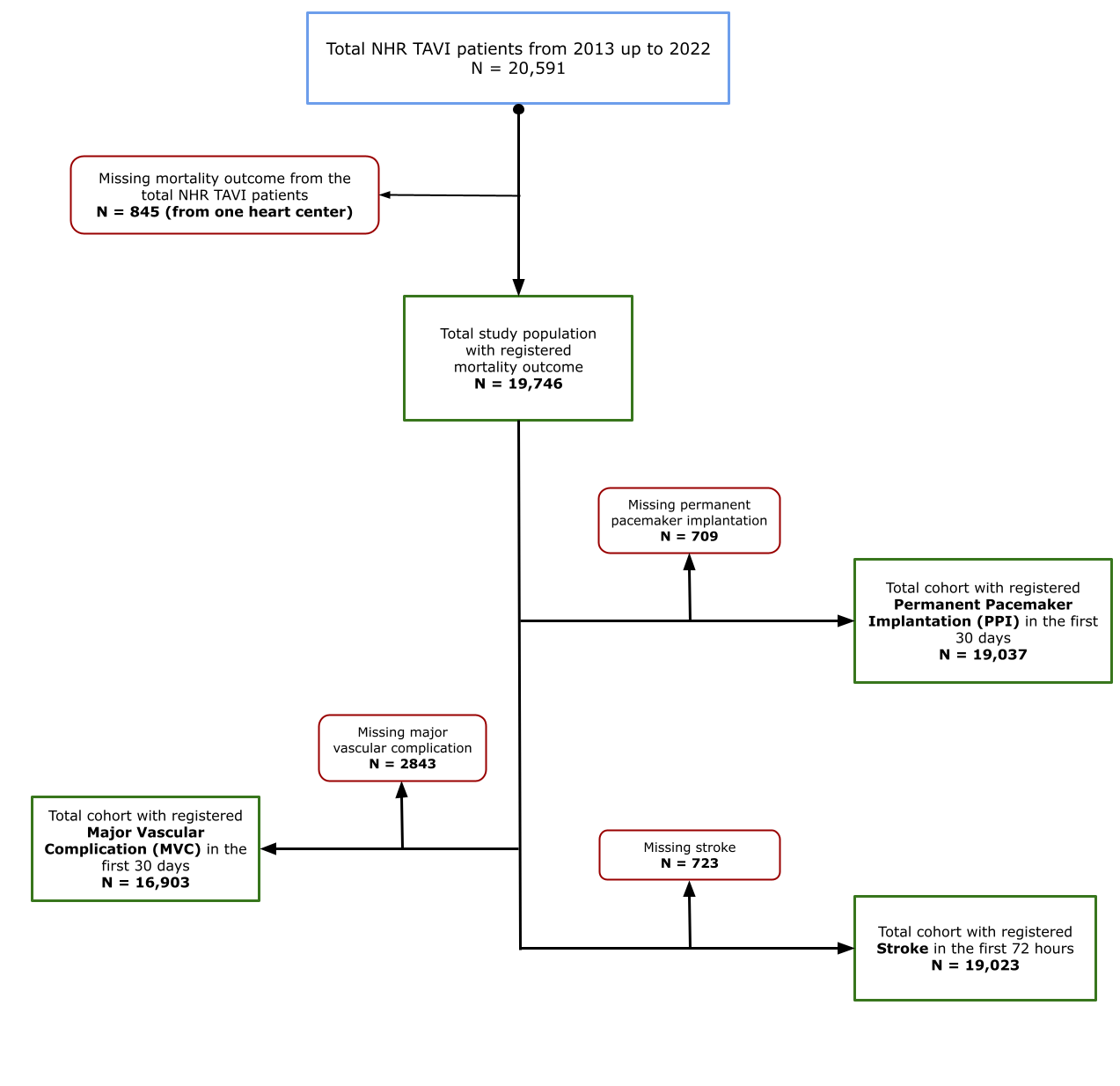


E-Figure 2. Annual (2013 up to 2022) MACE incidence in TAVI patients in the Netherlands stratified by sex. A. 30-day mortality, B. One-year mortality, C. Permanent pacemaker implantation, D. Major vascular complication, E. Stroke, and F. MACE.


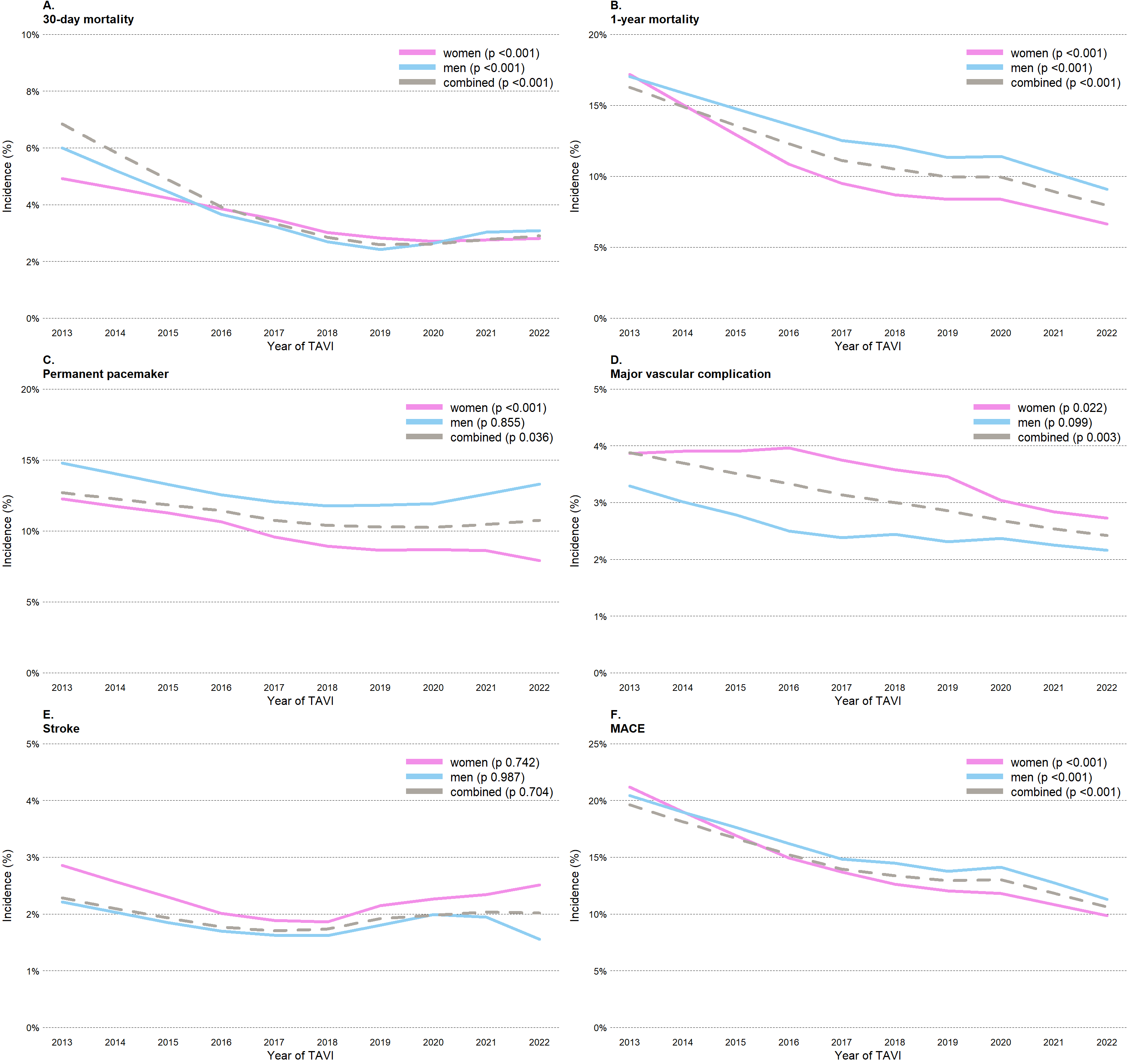


*p-values: significance of using calendar year of TAVI procedure as a linear predictor in the corresponding outcomes by a logistic regression model.

E-Figure 3. Incidence of each of the TAVI-related MACE outcomes per age (in years) for the 19,746 TAVI-patients (bottom 1.25 and top age 98.725 percentiles were trimmed).


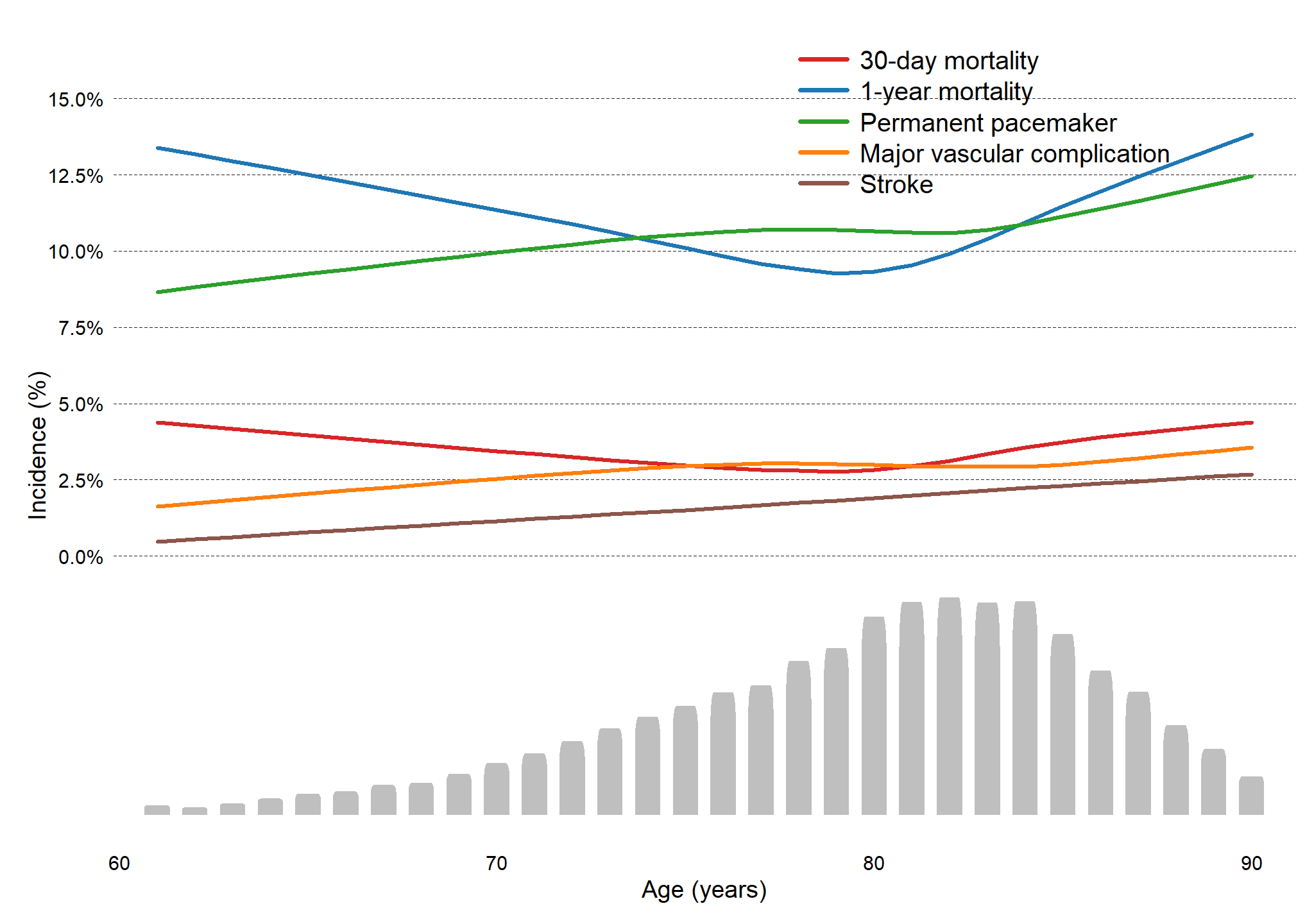
 Vertical bars at the bottom represent the proportion of patients belonging to the corresponding age in the dataset. Each bar represents one year of age.

# Appendix B

## Supplementary Methods

### Statistical analysis

For summarizing baseline patient characteristics, we used patient demographics, past medical history, and procedural details. Continuous variables were summarized as mean with standard deviation for the normally distributed variables, or as median with interquartile range otherwise. Categorical variables were summarized as frequencies and percentages. Baseline characteristics were summarized for the TAVI cohort in general, as well as for women and men separately.

After describing baseline characteristics, the incidence of each of the MACE outcomes was calculated. Then the incidence was analysed in five consecutive two calendar year periods (2013-2014, 2015-2016, 2017-2018, 2019-2020, and 2021-2022). A two-year interval was selected to balance the need for sufficient granularity and simplicity.

Next, the trends over the years were calculated. We tested the trends of the TAVI-related MACEs using the Cochran Armitage test for trends [1, 2].

Subsequently, we studied the incidence of each MACE in relation to patient age.
In this age analysis, two different approaches were used. First, by dividing patients into three fixed age groups: patients <75 years, between 75-80 years, and >80 years. Second, by using age as a continuous variable and plotting the incidence of each outcome versus age.

In both the calendar year, and patient age analyses, we further analysed MACE incidence by sex. We did so by repeating the steps of each analysis, with the addition of stratifying the TAVI patients based on sex.

A p-value <0.05 was considered significant for all analyses. All statistical analyses were performed in the R statistical environment version 4.4.1 [3].

## Supplementary Discussion

Little data exist on MACEs in various age groups and between sex differences among TAVI patients. Habertheuer et al., reported that TAVI patients with neurologic events tended to be older women (mean age 86 years, 69% women), compared to patients without neurologic events (mean age 82, 31% women) [4]. We observed a similar case with our analyses where women were found to have a higher incidence of stroke compared to men. In addition, we also found that regardless of age, women always had a higher incidence of stroke than men. Furthermore, this difference would increase with older age.
A recent study of Dabrowski et al. reported a significantly higher operative-risk EuroSCORE-II risk score of 7.2, and higher rate of 30‑day and 1‑year mortality (5% and 9.4%, respectively) in patients ≥85 years [5]. However, they reported similar rate of non-mortality MACEs in patients aged >85 years, except for MVC which were significantly higher in elderly patients. Other recent data from a large TAVI population showed that 3.9% of the patients that underwent transfemoral TAVI had developed MVC, where the majority of them were females [6].

Supplementary references

1. Cochran WG. Some Methods for Strengthening the Common Chi-squarred Tests. Biometrics. 1954;10(4):417-51.

2. Armitage P. Tests for Linear Trends in Proportions and Frequencies. Biometrics. 1955;11(3):375-86.

3. R Core Team. R: A Language and Environment for Statistical Computing. R Foundation for Statistical Computing,Vienna, Austria. 2019.

4. Habertheuer A, Gleason TG, Kilic A, et al. Sultan I. Impact of Perioperative Stroke on Midterm Outcomes After Transcatheter Aortic Valve Replacement. Ann Thorac Surg. 2020;110(4):1294-301.

5. Dabrowski M, Pylko A, Chmielak Z, et al. Witkowski A. Comparison of transcatheter aortic valve implantation outcomes in patients younger than 85 years and those aged 85 years or older: a single-center study. Pol Arch Intern Med. 2021;131(2):145-51.

6. Sherwood MW, Xiang K, Matsouaka R, et al. Rao SV. Incidence, Temporal Trends, and Associated Outcomes of Vascular and Bleeding Complications in Patients Undergoing Transfemoral Transcatheter Aortic Valve Replacement: Insights From the Society of Thoracic Surgeons/American College of Cardiology Transcatheter Valve Therapies Registry. Circ Cardiovasc Interv. 2020;13(1):e008227.
